# Emotional Embodiment in the Digital Age: The Digitization of Emotions

**DOI:** 10.3390/bs16040487

**Published:** 2026-03-25

**Authors:** Vincenzo Auriemma

**Affiliations:** Department of Political and Social Studies, University of Salerno, 84084 Salerno, Italy; vauriemma@unisa.it

**Keywords:** digitization of emotions, adolescence, emotional embodiment, artificial intelligence, empathy, digital wellbeing, sociology of emotions

## Abstract

The objective of this paper is to propose a sociological and interdisciplinary framework for analyzing the digitization of emotions in adolescence. This contribution aims to promote theoretical reflection and inform educational and political interventions in the digital age, framing adolescents’ digital experiences as emotionally embodied and socially integrated processes. These aspects are of paramount importance due to the rapid proliferation of digital technologies and artificial intelligence, which have precipitated a profound transformation in the emotional, relational, and educational experiences of adolescents. The role of digital and AI-based environments in mediating communication is expanding beyond the scope of simple facilitation. These environments are increasingly implicated in the production, modulation, and regulation of emotions, thereby influencing developmental trajectories and identity formation processes. This phenomenon is theorized as a socio-technical process, wherein emotions are embodied, narrated, and governed within digital environments. The article introduces the concept of digital emotional embodiment, drawing on the sociology of emotions, theories of embodiment, and critical perspectives on artificial intelligence. Specifically, the concept refers to the manner in which adolescents experience and express emotions through avatars, images, emojis, algorithmic feedback, and AI-mediated interactions. Therefore, it is imperative to underscore the evolution of empathy, which is progressively configured as a virtualized and datafied process, diverging from the tradition established by Hume and characterized by sympathy. In contemporary processes, shaped by the logic of platforms, recommendation systems, and emotionally reactive technologies, conventional emotional concepts have undergone deconstruction, and digital constructs are undergoing a gradual restructuring. In this context, AI systems do not merely reflect adolescents’ emotions but rather actively contribute to the construction of emotional narratives, influencing emotional regulation, social connection, and future orientation. Digital environments have been shown to encourage emotional expressiveness, experimentation, and inclusivity. Conversely, they have the capacity to encourage emotional standardization, dependency, and forms of affective vulnerability, particularly during a sensitive developmental stage such as adolescence.

## 1. Introduction

The term “adolescence” is used to denote the critical stage of development during which significant changes occur in terms of emotional regulation, identity formation, and social behavior (Steinberg, 2014; Sawyer et al., 2018). In contemporary societies, these processes are becoming increasingly evident in digitally mediated environments, characterized by the pervasive presence of mobile devices, social media platforms, and artificial intelligence (Boyd, 2014; Livingstone & Third, 2017). To date, behavioral and psychological research has extensively examined the effects of digital technologies on adolescents’ mental health, cognitive functions, and social behaviors. However, emotions are often considered individual outcomes, risk factors, or even innate elements of our behavior, rather than socially integrated processes mediated by culture and technology (Odgers & Jensen, 2020; Twenge et al., 2019). This tendency has the potential to obscure the role of digital environments in shaping emotional experience, as evidenced by research conducted by Lupton (2018b) and Illouz (1997). From a behavioral science perspective, digital technologies are often conceptualized as external stimuli that influence emotional responses, well-being, or maladaptive behaviors (Illouz, 1997). However, this stimulus-response framework proves inadequate to capture the complexity of adolescents’ emotional lives in digital and AI-driven contexts. Emotions are not exclusively induced by digital content; rather, they are increasingly produced, structured, and regulated through socio-technical systems that mediate interaction, visibility, and recognition (Lupton, 2018a; Belk, 2013). To elaborate, emotions are characterized by two distinct elements: culture and technological transformations. This facet assumes particular pertinence in the context of adolescents, whose emotional repertoires and identity narratives are undergoing a period of significant development and are thus particularly susceptible to environmental influences. A substantial body of research, including studies by Gall et al., has underscored the multifaceted opportunities and risks associated with adolescents’ digital engagement. On the one hand, there is an increase in emotional expressiveness, creativity, and social inclusion. Conversely, emotional dysregulation, addiction, and vulnerability have been identified as contributing factors (Gall et al., 2021). However, the majority of research in this domain concentrates on behavioral outcomes (i.e., the underlying motivations for actions), while dedicating minimal attention to the theoretical processes through which emotions are digitally structured (e.g., the mechanisms underlying the digitization of love). It must be acknowledged that there are crucial questions that have not been adequately addressed. These include the following: The following inquiry is posited: How are emotions represented when interactions are mediated by interfaces, avatars, and algorithms? A critical examination of the implications of heightened empathy, precipitated by platform logic and AI-driven feedback, is imperative to inform a nuanced understanding of its potential consequences. The subsequent inquiry pertains to the impact of these transformations on the emotional development of adolescents over time. In order to address the aforementioned inquiries, the present paper will commence with concepts related to the digitization of emotions, understood here as a socio-technical process through which emotions are increasingly embodied, narrated, quantified, and governed within digital environments (Auriemma, 2023a). Rather than emphasizing emotional expression, the digitalization of emotions necessitates an examination of the infrastructural and symbolic conditions that influence emotional experience. Consequently, digital platforms and Artificial Intelligence systems do not merely serve as environments for emotional interactions; rather, they actively determine their visibility, social recognition, and marginalization. This, in turn, influences emotional learning and regulation. At the core of this process is the transformation of emotional embodiment (Niedenthal et al., 2009). In this study, the concept of digital emotional embodiment is introduced as a means of describing the ways in which emotional experiences are increasingly shaped and enacted within technologically mediated environments. In this article, digital emotional embodiment is defined as a socio-technical process through which emotional experience is co-constituted across three analytically distinct but interrelated dimensions: (1) embodied affective states, rooted in bodily perception and physiological activation; (2) mediated expression, enacted through digital interfaces such as avatars, emojis, and platform-specific communicative forms; and (3) algorithmic modulation, whereby emotional signals are captured, quantified, and reoriented through data-driven systems. This framework is analytically bounded in that it does not refer to all forms of digitally mediated emotion but specifically to those in which emotional experience is simultaneously embodied, externally represented, and recursively shaped by computational infrastructures. In this sense, digital emotional embodiment differs from related constructs such as emotional expression or online affect, as it foregrounds the recursive interplay between bodily processes, symbolic mediation, and algorithmic feedback. What this framework uniquely adds is a relational understanding of emotional experience as simultaneously embodied, mediated, and algorithmically governed, rather than treating these dimensions as analytically separate. Contrary to the assumption that digital environments engender a disembodied form of emotional interaction, this concept underscores the notion that emotional life persists as a profoundly embodied phenomenon, concurrently becoming technologically mediated. In this sense, emotions emerge from the dynamic interplay between bodily experience, social interaction, and digital infrastructures that structure communication and recognition processes. The concept of digital emotional embodiment underscores the manner in which emotional expression and regulation are reconfigured within contemporary socio-technical ecosystems, particularly among adolescents whose identity formation and social interactions are increasingly shaped by digitally mediated contexts. This phenomenon can be attributed to the long-standing theoretical framework positing emotions as embodied phenomena, underpinned by bodily perception and sensorimotor processes. However, in digital contexts, embodiment is increasingly mediated by technological interfaces that extend, reshape, or partially replace bodily presence. In this regard, adolescents (and often adults) employ avatars, images, emojis, and algorithmic feedback to express their emotions, thereby giving rise to forms of digital emotional embodiment that blur the boundaries between body, technology, and social interaction (Zimmermann et al., 2023). Hybrid forms present a significant challenge to long-standing assumptions in behavioral sciences. These assumptions pertain to fundamental concepts such as authenticity, immediacy, and emotional regulation. The evolution of empathy signifies a pivotal facet of adolescent emotional maturation within AI-mediated contexts. Empathy is traditionally conceived as an intersubjective capacity rooted in embodied resonance and face-to-face interaction. However, it is increasingly shaped by digital infrastructures that translate emotional signals into data, metrics, and recommendations. In this context, empathic processes are partially virtualized and transformed into data, influenced by platform architectures and emotionally responsive technologies (Scribano & Mairano, 2021). AI systems, in particular, play an active role in organizing emotional narratives, prioritizing certain content, interactions, and affective styles. This contributes to the emotional regulation and social orientation of adolescents. Despite the evident increase in the importance of these dynamics, as will be demonstrated in the following brief bibliographic review, current behavioral research lacks a comprehensive analysis that integrates emotions, embodiment, and AI into a unified analytical perspective. In the absence of a comprehensive theoretical framework, there is a risk of reducing complex emotional transformations to symptoms or behaviors at the individual level. This would result in the neglect of the broader socio-technical processes that are in operation. This deficit is especially salient in educational and well-being contexts, where interventions tend to prioritize emotional skills without acknowledging the environmental influences that shape emotional experience. A comprehensive literature review was conducted using the Bibliometrix 5.0 software to analyze the volume of publications by discipline and topic. Furthermore, it critically examines the enabling and limiting effects of digital environments, highlighting how these can foster emotional expression and inclusion while simultaneously promoting emotional standardization, addiction, and emotional vulnerability. In this context, the issue of adolescents’ emotional well-being in the digital age will be addressed. It is imperative to adopt an approach that transcends individual interventions; instead, the development of digital emotional literacy and AI governance strategies must be promoted. The present study aims to contribute to the theoretical debate on the embodiment of emotions in digital contexts by reframing emotions as socially and technologically integrated processes. Furthermore, the study seeks to inform educational and policy-oriented approaches to adolescent development.

## 2. Theoretical Background: Emotions, Digitalization and Embodiment

The present article addresses a particular lacuna concerning the intersection of behavioral sciences, the sociology of emotions, and digital studies. Despite the fact that a substantial body of behavioral research has been dedicated to examining the psychological outcomes of digital media use in adolescence, including anxiety, stress, and emotional regulation, these studies frequently conceptualize emotions as individual-level outcomes. Consequently, there is a paucity of attention devoted to the socio-technical environments that structure emotional experience. Concurrent with this, sociological and critical studies of digital platforms have examined processes of datafication, algorithmic governance, and platform power. However, these studies have seldom integrated these insights into a systematic description of emotional development in adolescence. Therefore, the following article will attempt to establish a connection between the two studies by proposing digital emotional embodiment as a conceptual tool for analyzing the process of acquiring, manifesting, and regulating emotions within AI-mediated environments. Accordingly, the present study reorients the analytical focus from the effects of digital technologies on emotions to the conditions under which emotional dynamics are constituted in contemporary digital societies. Additionally, digital emotional embodiment is proposed as an analytical and critical framework, rather than a purely descriptive label. From an analytical perspective, it is employed to examine how emotional experience is structured through the interaction between embodied sensations, mediated interaction, and algorithmic feedback. From a critical vantage point, it underscores the power relations inherent in digital infrastructures that determine which emotions are made visible, valuable, or governable. The concept does not imply that emotions become disembodied in digital contexts; on the contrary, it emphasizes how embodiment is reconfigured through technological mediation, producing new forms of emotional attunement, regulation, and recognition that are neither completely online nor offline, but structurally hybrid. As previously mentioned, in the domain of behavioral and psychological sciences, emotions are frequently conceptualized as internal states that are linked to cognitive evaluations, physiological responses, and observable behaviors. While these approaches have yielded significant insights into emotional functioning, they have been observed to prioritize individual mechanisms, often overlooking the social and cultural dimensions through which emotions are imbued with meaning. From a sociological perspective, emotions are not only individual experiences, but relational and socially rooted processes, shaped by norms, symbols, and interactional contexts (Hochschild, 1979; Kemper, 1978; Turner & Stets, 2005). The field of sociology has long emphasized that emotions are learned, regulated, and expressed within specific social contexts. Emotional experience is therefore inseparable from the social structures and cultural contexts that define which emotions are appropriate, how they should be expressed, and how they are interpreted by others (Hochschild, 1979; Kemper, 1978; Turner & Stets, 2005). This perspective is particularly salient during adolescence, a developmental stage during which the emotional repertoire is actively negotiated through interaction with peers, social recognition, and identity construction. In the context of digitally mediated environments, these processes do not cease to exist; rather, they undergo a reorganization (Turner & Stets, 2005). Digital platforms engender novel emotional norms, feedback mechanisms, and forms of visibility that exert influence on emotional expression and recognition. Algorithmic reactions, comments, and recommendations, although ostensibly social signals, have been demonstrated to guide emotional behavior, thereby reinforcing certain affective styles while marginalizing others. Consequently, emotions do not exclusively originate in interpersonal interaction; they also originate in platform-mediated social regulation, which is assuming an increasingly central role in the emotional lives of adolescents. The concept of digitization entails more than merely transposing existing practices into digital formats; it encompasses a comprehensive process of socio-technical reorganization within the context of emotions. Digitization, therefore, entails a profound transformation in the manner in which emotions are produced, communicated, evaluated, and governed within digital environments. Contrary to the prevailing notion that digital technologies are external to emotional life, they have been shown to actively participate in it, shaping the possibilities for interaction and affective expectations. As posited by several scholars, including Belk and Lupton, the role of digital platforms extends beyond mere hosting or facilitation of emotional expression. These platforms have been shown to function as critical infrastructures of emotional life, actively contributing to the organization and regulation of emotions (Belk, 2013; Lupton, 2018a). From this perspective, platforms function as socio-technical environments that shape the visibility of emotions, direct attention, and mediate social recognition through algorithmic systems that select, amplify, or marginalize specific forms of emotional expression (Auriemma, 2025). Emotional visibility is never neutral; rather, it is contingent upon the logics of each platform, which prioritize certain content based on criteria such as engagement, affective intensity, and circulability. This phenomenon is exemplified by prominent social media platforms like Instagram and TikTok. Emotions that are readily identifiable, communicable, and quantifiable are often accorded greater value, while those that are more nebulous, intricate, or challenging to translate into standardized signals are susceptible to remaining imperceptible (Auriemma, 2025). In this regard, platforms not only mirror users’ emotional states but also serve to determine which emotions are deemed socially significant and meriting consideration. In the context of social media, emotions are increasingly intertwined with data flows, metrics, and predictive systems, thereby giving rise to processes of datafication of emotional experience. Likes, reactions, comments, viewing times, and interaction patterns function as quantitative indicators of emotional intensity and value, translating complex affective states into measurable signals (Auriemma, 2025). Subsequently, these data are processed by algorithmic systems that not only record emotions but also utilize them to anticipate behavior, personalize content, and guide future interactions. In this context, emotions assume a dual nature: Firstly, they persist as subjective experiences that are inherently embodied. Secondly, they transform into entities that can be calculated and predicted, thereby becoming incorporated into feedback loops that influence the manner in which they are experienced and expressed. This phenomenon carries profound ramifications, particularly for adolescents, as their emotional learning and social recognition are shaped by environments where emotional value is perpetually mediated by metrics and algorithms. Digital platforms have been demonstrated to influence not only the expression of emotions but also emotional expectations, criteria for affective validation, and modes of emotional regulation (Bucher, 2018). This perspective enables us to transcend a mere instrumental perspective on technology, acknowledging its profound influence in shaping contemporary emotional experiences (Bucher, 2018). This assertion is bolstered by the mounting evidence that emotions do not merely traverse digital media, but rather, they are increasingly embedded within socio-technical systems, thereby influencing their form, visibility, and meaning. This integration of emotions into digital systems has the potential to profoundly redefine the very conditions of emotional life in the digital landscape. Consequently, researchers and scholars, including Bucher, Lupton, and Scribano, are confronted with a conception of “emotional embodiment” that is amplified compared to the past and detached from the neurosociological logic of the American tradition (Auriemma, 2025). In accordance with Scribano’s assertion, it can be posited that emotional expressions are captured, quantified, and subsequently returned to users in ways that exert an influence on subsequent emotional behavior. This dynamic is particularly evident in the case of adolescents, whose emotional development takes place in environments that continuously monitor and respond to affective signals. A longitudinal analysis of the emotional responses exhibited by French youth under the age of 15 could offer a compelling avenue for future research. A comparative study of this data with that of other European youth within the same age bracket could provide valuable insights. This study’s findings are particularly pertinent in light of the French National Assembly’s approval of a bill in January 2026, which aims to restrict children under the age of 15 from accessing social networks. The bill, currently under review in the Senate, aims to implement the law in September 2026 (Assemblea Nazionale Francese, 2025). In light of the prevailing context, marked by phenomena of media integration and mediation, the digitalization of emotions can be construed as a socio-technical process, whereby emotions are progressively incorporated into digital frameworks. Consequently, emotions are not merely expressed in digital media; they are also shaped by the logic of platforms that prioritize immediacy, visibility, and engagement (Beer, 2017). These dynamics contribute to the standardization of emotional expression, while allowing new forms of experimentation and creativity to emerge. It is imperative to comprehend this tension to facilitate a thorough analysis of the opportunities and risks associated with adolescents’ digital engagement. A foundational premise underpinning theories concerning digital emotions is that they are embodied phenomena, inherently intertwined with bodily sensations, perception, and action. Theories of embodiment underscore the notion that emotional experience is not confined to cognitive representations but rather emerges from the dynamic interaction between the body and the environment (Niedenthal et al., 2009). However, the advent of digital environments has introduced a paradigm shift in conventional conceptions of embodiment. Technological mediations, such as virtual reality headsets, extend or partially replace physical presence, challenging established notions of embodiment. Adolescents engage in emotional interaction through the use of avatars, images, emojis, and text messages, which function as proxies for bodily expression. The use of mediated forms of embodiment does not negate the body; rather, it redefines its role, thereby generating hybrid experiences in which emotional meaning is co-constructed by bodily sensation and technological mediation (Auriemma, 2023b). Research conducted on virtual environments and avatar-based interaction, such as those by Gall et al., suggests that digital embodiment can intensify emotional responses, influence self-perception, and shape social behavior (Gall et al., 2021; Zimmermann et al., 2023). In the case of adolescents, these processes are inextricably linked with the formation of identity. The notion of the “Meadian self” emerges in this context, signifying that digital embodiment serves as a milieu for the exploration of emotional expression and social roles (Mead, 1934). Consequently, the phenomenon of emotional embodiment in digital environments must be conceptualized not as a diminution of authenticity, but rather as a metamorphosis of the conditions under which emotions are experienced and recognized. Conversely, empathy, while still operating within the domain of emotionality, has been conceptualized as an intersubjective capacity founded on embodied resonance and face-to-face interaction. However, the neuroscientific tradition has endeavored to situate it within biological constructs that do not fully capture its essence (Zimmermann et al., 2023). While these approaches have yielded substantial outcomes, they have the tendency to underestimate the role of social and technological mediation in shaping empathic processes. Consequently, empathy can be conceptualized as an element that is culturally learned, modeled, and transformed over time. The reference in question may be attributable to a variety of factors, including but not limited to specific national leaders, manifestations of nationalisms, and populisms. However, in digital environments, empathy is increasingly mediated by platforms and artificial intelligence systems that structure the way emotional signals are presented and interpreted. Algorithmic systems have been demonstrated to exert a substantial influence on the visibility of emotional narratives, privileged interactions, and the representation of emotional responses. Consequently, empathy is partially virtualized and transformed into data, embedded in socio-technical systems that guide emotional attention and engagement (Scribano & Mairano, 2021). From this perspective, the concept of empathy can be considered in a different form, one that is culturally distant from the familiar form but conceptually linked to the same “purpose.” In this transformation, artificial intelligence plays a particularly significant role. Artificial intelligence-based systems have been shown to respond to users’ emotions and actively shape emotional interaction by predicting preferences, recommending content, and simulating emotional responsiveness. Notwithstanding the substantial progress made by behavioral sciences in identifying the emotional outcomes associated with the use of digital technology, a more complete understanding of the phenomenon requires consideration of the socio-technical conditions that shape the emotional experience.

## 3. Materials and Methods

**Figure 1:**
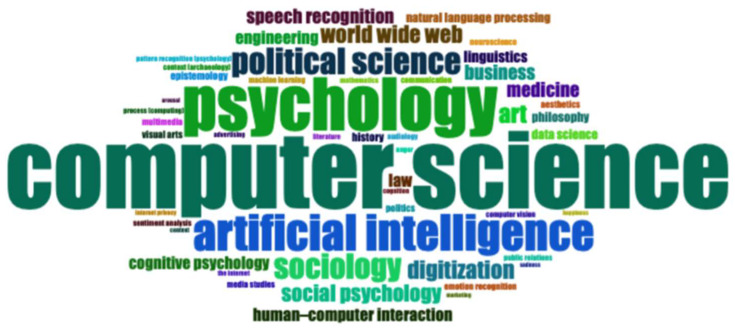
World Cloud by subject area. Source: Bibliometrix.

Despite the predominantly theoretical and conceptual orientation of this article, it incorporates bibliometric analysis as a complementary methodological strategy. Bibliometric mapping is a useful tool for contextualizing the interdisciplinary debate within which this conceptual proposal is situated. In this context, interdisciplinarity is the only theoretical framework that can adequately address the phenomenon of the digitization of emotions in adolescence. This framework focuses on emotional embodiment and empathy in AI-mediated environments. The decision was made to prioritize interdisciplinarity, as transdisciplinarity, which is more meaningful and appropriate, is challenging to implement at this particular juncture in history. This approach is predicated on the utilization of the foundational concepts from each discipline, encompassing the comprehension of these notions and assumptions and subsequently rendering them malleable to a univocal conception, a common dictionary, and a language aimed at universal comprehension (Nicolescu, 2002; Klein, 2010). Therefore, by integrating the sociology of emotions, embodiment theory, and critical studies on AI, this work endeavors to synthesize and broaden existing behavioral research, proffering conceptual tools to enhance the comprehension of the emotional development of adolescents in digital contexts. The objective of this analysis is not to provide empirical validation of the proposed framework, but rather to systematically map the intellectual landscape in which research on digital emotions, embodiment, and adolescence currently resides. From an epistemological perspective, bibliometric analysis is employed as a reflective tool that allows for the identification of dominant research clusters, thematic emphases, and disciplinary boundaries within the existing literature. This approach lends support to the theoretical argument by underscoring structural patterns in knowledge production, encompassing conceptual fragmentation and disciplinary targets. These elements collectively motivate the necessity for an integrative framework, such as digital emotional embodiment. Therefore, based on the extant findings within the historical and social context, and prior to undertaking an in-depth theoretical analysis, it is deemed necessary to conduct a systematic review of the extant literature through bibliometric analysis. The objective of this clarification is to establish the context for the current contribution within the pertinent field of study. As demonstrated, the intricacies of emotions, empathy, and emotional embodiment preclude the establishment of universal principles. Nevertheless, the establishment of a shared language among disparate disciplines stands as the objective of our interdisciplinary discourse. To this end, we employed Bibliometrix tools, namely OpenAlex, which is integrated as a library in the RStudio environment (V 2026.01.0-392). This integration facilitates the examination of scientific output in a structured manner using keyword-based queries (Aria & Cuccurullo, 2017). A query containing the following keywords was used to implement a quantitative methodological approach: The following terms are to be defined for the purpose of this study: “digitization of emotions,” “adolescence,” “emotional embodiment,” “artificial intelligence,” “empathy,” “digital wellbeing,” and “sociology of emotions.” The selection of these keywords reflects the interdisciplinary ambition of the study, while recognizing that bibliometric methods favor explicit terminological convergence and may underrepresent conceptually relevant works that do not adopt standardized labels. The time period (2015–2026) was selected to encompass both pre-pandemic and post-pandemic academic output, with the understanding that the year 2026 was excluded due to the timing of the research, which was conducted in February of that year. To this end, the tools provided by Bibliometrix were utilized, with a particular focus on OpenAlex, which is integrated as a library within the RStudio environment. This tool facilitates the structured analysis of scientific output through keyword-based queries (Aria & Cuccurullo, 2017). To this end, we employed a quantitative methodological approach, utilizing a query comprising the following keywords: The following terms are key to understanding the subject under discussion: “digitalization of emotions,” “adolescence,” “emotional embodiment,” “artificial intelligence,” “empathy,” “digital well-being,” and “sociology of emotions.” The selection of these keywords reflects the study’s interdisciplinary ambition, while acknowledging that bibliometric methods favor explicit terminological convergence and may underrepresent conceptually relevant works that do not adopt standardized labels. The time period (2015–2026) was selected to encompass both pre-pandemic and post-pandemic academic output, with the understanding that results for the year 2026 are not available as the research was conducted in February 2026. The corpus of analyzed publications was compiled through systematic searches of major academic databases, including Scopus and Web of Science. The selection of publications was executed through a meticulous combination of keywords pertaining to emotions, digital environments, artificial intelligence, empathy, embodiment, and adolescence (e.g., “digital emotions,” “emotional AI,” “digital empathy,” “emotional embodiment,” “adolescents and digital media”). The present study exclusively incorporates peer-reviewed journal articles written in English. In order to examine possible shifts in the conceptualization of digitally mediated emotions, the corpus was divided into two temporal subsets: publications produced before the onset of the pandemic (up to 2019) and those published during and after the pandemic period (from 2020 onward). This distinction enabled us to investigate whether the pandemic context contributed to shifts in thematic emphasis and terminological convergence within the field. The resulting dataset was then analyzed using bibliometric mapping techniques to identify patterns of co-occurrence among keywords and to pinpoint the main thematic clusters emerging in the literature. While this approach enables a systematic overview of recent trends, it is important to note its limitations. Bibliometric analyses are inherently constrained by database coverage, keyword dependency, and publication biases. These analyses tend to favor quantity-oriented disciplines over interpretive or critical traditions. Consequently, the findings should not be construed as a comprehensive representation of the field; rather, they should be regarded as an indication of the prevailing epistemic orientations within mainstream scientific production. A thorough analysis of the database yielded a total of 371 contributions, comprising 230 scientific articles and 141 books and book chapters. A salient initial figure pertains to the Annual Growth Rate, which is equivalent to −10.76%. This finding suggests a diminution in the collective interest surrounding the subject. This indicator could be interpreted as a sign of saturation in the field, as if certain theoretical and applied perspectives were now considered well established. However, a particularly salient figure merits attention, as it engenders an alternative interpretation of the data in this study. The Document Average Age, which is equivalent to 4.93 years, signifies that a substantial proportion of recent literary production tends to perpetuate relatively stable theoretical assumptions, frequently regarded as invariants, without conducting sufficient comparison with the profound social, cultural, and technological transformations that are substantially redefining the modes of emotional interaction. A bibliometric analysis reveals a predominance of computational sciences and artificial intelligence, despite the presence of interdisciplinary contributions that favor theoretical and applied integration between technological, psychological, and socio-organizational dimensions. However, this interdisciplinarity appears to be predominantly confined to the so-called hard sciences, while approaches that address the digitization of emotions from a cultural and social perspective are marginal. Contributions explicitly related to the field of sociology are limited (approximately ten publications), thereby confirming a significant underrepresentation of the sociology of emotions in the dominant scientific debate. As illustrated in Figure 1 (word cloud), which offers a synoptic representation of the predominant thematic domains that emerged from the analysis, there is a marked centrality of computer science and artificial intelligence, which serve as the structuring domains of the field. These poles are flanked by contributions from the social and human sciences, particularly psychology, sociology, and social psychology, thereby confirming the formally interdisciplinary nature of the sector. The presence of terms such as “human-computer interaction,” “speech recognition,” “data science,” and “digitization” indicates a strong application and technology orientation. References to medicine and law suggest a progressive extension of artificial intelligence into regulatory and healthcare areas.

**Figure 2:**
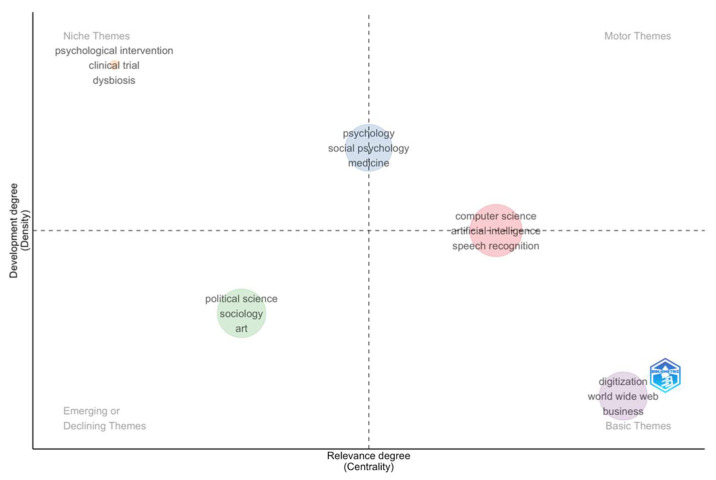
*Thematic map*. Source: Bibliometrix.

A more in-depth analysis of the conceptual structure of the field is provided by the thematic map (Figure 2), which distributes thematic clusters according to their degree of centrality (relevance) and density (level of development). The cluster pertaining to computer science, artificial intelligence, and speech recognition falls within the scope of motor themes, signifying that these topics are not only extensively developed but also profoundly interconnected, playing a pivotal role in literary production. The cluster composed of psychology, social psychology, and medicine occupies an intermediate position, suggesting an established area of research that is still in the process of systematic integration with the dominant technological trends. In the basic theme’s quadrant, the concepts of digitization, the World Wide Web, and business emerge, are widely used, and are characterized by high centrality but low density. These concepts configure themselves as cross-cutting and conceptually unstructured categories. Conversely, the cluster comprising sociology, political science, and art is identified as an emerging or potentially declining theme, as it is characterized by limited centrality and a low degree of internal development. Finally, the presence of niche themes, such as psychological intervention, clinical trials, and dysbiosis, signifies the existence of highly specialized domains that exhibit robust internal cohesion but exhibit marginal integration within the overarching framework of the field.

**Figure 3:**
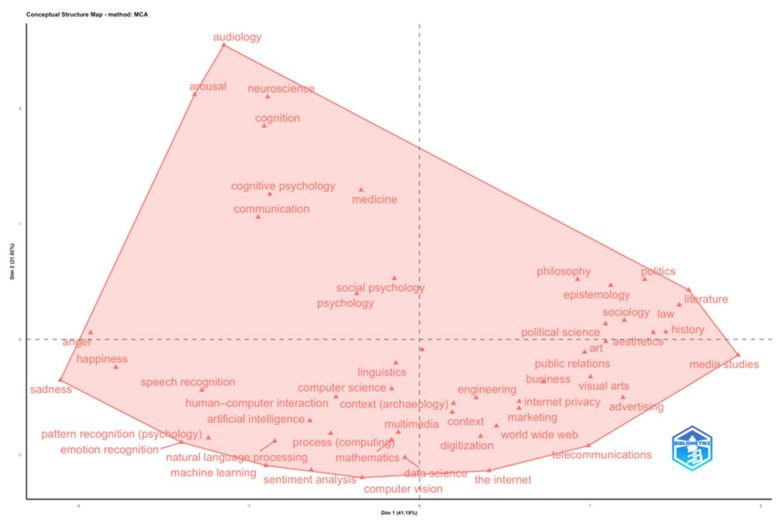
Conceptual structure map. Source: Bibliometrix.

To corroborate these dynamics, a factor analysis was executed using Multiple Correspondence Analysis (MCA), as illustrated in Figure 3. This analysis permitted the semantic configuration of the research field to be investigated. The conceptual map illustrates a pronounced dichotomy between a technological-computational sector, which is typified by terms such as “artificial intelligence,” “machine learning,” “computer science,” and “natural language processing,” and a socio-humanistic sector, encompassing “sociology,” “philosophy,” “political science,” and “law.” The disciplines that fall between these two extremes include psychology, social psychology, and medicine. This suggests that there is a role for conceptual mediation between technological and interpretative approaches.

The analysis, in its entirety, corroborates the multidisciplinary character of the field. However, it simultaneously underscores a structural disparity that favors the domain of computational sciences over socio-cultural perspectives. The interdisciplinarity observed principally pertains to the “hard” sciences, while sociological methodologies remain marginal, despite their explanatory potential in understanding the emotional transformation processes underway. In view of these findings, it is imperative to undertake a more profound exploration of the digitization of emotions as a socio-cultural and socio-technical phenomenon. This exploration must acknowledge the inextricable interconnection between emotional processes and the technological infrastructures, cultural contexts, and social dynamics that give shape to them. Consequently, this necessity constitutes the theoretical and methodological foundation of this contribution. In this sense, bibliometric analysis is not conceived as an end in itself; rather, it functions as a methodological bridge between existing research trends and the theoretical intervention posited in this article.

## 4. The Digitization of Emotions as a Socio-Technical Process

The bibliometric results examined in the previous paragraph corroborate the central hypothesis of this article, namely that certain sciences and concepts predominate. Indeed, the significant prevalence of themes pertaining to computer science, artificial intelligence, and psychological research suggests that emotions in digital environments are predominantly conceptualized as measurable signals, cognitive states, or behavioral outcomes. Conversely, the marginal positioning of sociology and adjacent disciplines suggests a persistent theoretical underestimation of emotions as socially integrated, embodied, and relational processes. This asymmetry is not merely descriptive but also has implications for theoretical perspectives. The prevailing trend in the extant literature, oriented towards individual and computational approaches, appears to neglect the role of socio-technical environments in the organization of emotional experience, particularly during adolescence. The concept of digital emotional embodiment directly addresses this gap by offering a framework that integrates behavioral insights with sociological analyses of platforms, algorithms, and emotional norms. In light of the findings from the preliminary research conducted, particularly in light of the interdisciplinary investigation focused on specific disciplines, it is imperative to acknowledge the pressing need for a comprehensive reinterpretation of emotions, especially in the contemporary era. This necessitates a thorough examination of the digitization of emotions as a socio-cultural phenomenon. The notion of emotional digitization should not be construed as merely an augmentation in the scope of emotional expression through digital media or as a straightforward translation of interactions from offline to online. Rather, it should be regarded as a transformation in the medium of communication, not a mere replication of forms from one context to another (Auriemma, 2025). Conversely, this process can be conceptualized as a socio-technical phenomenon, entailing the production, organization, and regulation of emotions within digital infrastructures, as opposed to their mere reproduction according to conventional models (Couldry & Hepp, 2017; Kitchin, 2014). In this context, emotions are intricately intertwined with technological systems that structure interaction, visibility, and feedback mechanisms, thereby helping to reshape the ways in which emotional experiences are constituted and socially recognized (Scribano & Mairano, 2021). From a sociological perspective, emotions are inherently linked to specific social contexts and are profoundly influenced by the structural conditions that give rise to them (Hochschild, 1979; Turner & Stets, 2005). In the context of digitally mediated environments, adolescents learn not only to express their emotions, but also to recognize which emotional states gain visibility, approval, or algorithmic amplification. The digitization of emotions can be interpreted as a form of emotional socialization embedded in socio-technical systems, in which affective recognition is closely linked to platform logic (van Dijck et al., 2018). A distinctive aspect of this process pertains to the quantification of emotions, which is conceptualized as the conversion of emotional expressions into quantifiable data (Kitchin, 2014). These emotions manifest as quantifiable signals, including actions such as clicks, reactions, emojis, and interaction patterns, which are processed by algorithmic systems. This statement does not intend to criticize the models of emotion measurement used in digital research. Rather, it aims to reflect on the perception that adolescents develop with regard to the instrumental use of emotions in such contexts. Algorithmic systems have been shown to influence the emotional ecosystem by prioritizing specific content, interactions, and affective styles, thereby contributing to the definition of what is emotionally relevant. Algorithmic mediation does not merely reflect users’ emotional states; rather, it actively influences their formation. Specifically, recommendation systems regulate emotional exposure by favoring content characterized by high intensity, immediacy, and engagement (Bucher, 2018). The dynamics of social media platforms such as Instagram or TikTok demonstrate the impact of recurrent exposure to algorithmically curated emotional stimuli on adolescents’ emotional regulation, attention, and social comparison processes. This exposure can reinforce specific emotional models that are regarded as “appropriate” or “desirable” within the digital environment over time. From this perspective, digital platforms can be conceptualized as emotional architectures, that is, systems capable of organizing and modulating the affective experience of users (Papacharissi, 2015). Emotions are subject to continuous feedback loops, in which expression, data collection, and algorithmic response are closely interconnected. This configuration calls into question the conventional distinction between emotional expression and emotional regulation. The latter appears to occur with increasing frequency through interaction with socio-technical systems, rather than exclusively through individual self-control. Digital environments are not neutral spaces; rather, they are contexts structured by platform logics that define dominant emotional norms (Gillespie, 2018). These logics favor emotions that are easily identifiable and readily shareable, such as enthusiasm, indignation, or excitement, while emotions that are more ambivalent or complex in nature tend to receive less visibility. The process of digitization of emotions has been shown to contribute to the standardization of emotions. This phenomenon occurs as users adapt their affective behavior to the technological affordances and social expectations embedded in various platforms. Concurrently, digital environments furnish spaces for emotional experimentation, particularly for individuals who may encounter forms of marginalization in offline contexts, as evidenced by the alternative expressive practices observable on platforms such as Twitch (Taylor, 2018). This ambivalence underscores the necessity to examine emotional normativity as a socio-technical phenomenon, embedded in design choices, algorithms, and interfaces, rather than as a mere consequence of social interaction. The digitization of emotions not only impacts the manner in which emotions are expressed, but also gives rise to novel forms of emotional governance. As demonstrated in the extant research, platforms employ a variety of mechanisms—including moderation policies, recommendation systems, and emotionally responsive technologies—to actively contribute to the regulation of affective environments (Beer, 2017). The role of artificial intelligence in this process is paramount, as it involves the analysis of emotional data to predict preferences, optimize engagement, and personalize content. For adolescents, whose emotional and empathic development remains in flux, such dynamics have the potential to influence emotional autonomy and psychological well-being (Steinberg, 2014). Therefore, based on the analysis conducted thus far, it can be posited that the digitization of emotions constitutes a structural element of contemporary adolescence, with the capacity to influence emotional experience within hybrid contexts characterized by profound interconnection between online and offline dimensions. It is therefore imperative to overcome the dichotomies between the digital and non-digital emotional spheres to comprehend how adolescents develop their emotional skills within socio-technical ecosystems that are simultaneously enabling and limiting. In this regard, it is imperative to underscore the pivotal role of symbolic interaction processes, particularly those facilitated by digital forms, in the analysis of emotional processes within the online realm during this historical period.

## 5. Digital Emotional Embodiment in Adolescence

In order to comprehend the emotional development of adolescents in the digital age, it is necessary to reinterpret the concept of embodiment, incorporating the influence of socio-technical media. Embodiment theories posit that emotions are processes based on bodily perception and sensorimotor engagement with the environment (Niedenthal et al., 2009). Recent contributions within the sociology of emotions further underscore the importance of examining emotional processes across multiple social levels. In particular, Hochschild’s recent reflections emphasize the operation of emotions at the individual, socio-structural, and political levels, shaping not only interpersonal interactions but also broader public and political dynamics. In contemporary digital societies, emotional narratives are increasingly disseminated through online media environments. In these environments, political actors strategically mobilize emotions to influence public opinion and social behavior. This perspective underscores the importance of examining emotional life within digitally mediated ecosystems, where emotional expression, recognition, and circulation are intricately linked with technological infrastructures. Likewise, Turkle’s research on relationships mediated by technology offers significant insights into the evolution of empathy and emotional interaction in digital environments. Turkle (2017) posits that digital communication technologies have the capacity to reconfigure the conditions under which individuals experience intimacy, empathy, and social connection. Adolescents, in particular, are subject to a unique set of challenges and opportunities presented by interactions with digital platforms and emerging AI systems. These interactions may result in the emergence of new forms of emotional engagement that are characterized by mediation, fragmentation, and continuous connectivity. Engaging with these perspectives facilitates the conceptualization of the notion of digital emotional embodiment within a broader sociological discourse on the reconfiguration of emotional experience in technologically mediated environments. In the context of digitally mediated environments, the body does not disappear, but rather undergoes an extension and reconfiguration through the mediums of screens, interfaces, and symbolic systems that serve as mediators of affective experience (Farman, 2012; Hansen, 2006). These forms of digital embodiment extend beyond merely reproducing offline emotional expression, but rather introduce novel modes of affective experience. These modes are distinguished by immediacy, persistence, public visibility, and continuous feedback (Gall et al., 2021). The efficacy of these modes of embodiment in the context of social interaction in digital environments has been demonstrated. Their ability to facilitate the establishment and sustenance of affective relationships in mediated environments is well-documented. However, the intensive and normatively oriented use of these technologies has the potential to generate problematic dynamics, especially when emotional expression is systematically integrated into the logic of economic valorization. In such circumstances, digital emotional expression runs the risk of being absorbed into forms of emotional capitalism, in which affectivity becomes a monetizable and performative resource, as can be observed in specific platformed contexts, including OnlyFans (Illouz, 1997). In such contexts, the digitized emotional body is exposed, measured, and evaluated according to parameters of visibility and desirability, thereby redefining the boundaries between intimacy, emotional labor, and social recognition. Digital emotional embodiment assumes particular pertinence during adolescence, a developmental period distinguished by heightened sensitivity to social evaluation and peer recognition (Steinberg, 2014). Digital environments serve to amplify these dynamics, thereby rendering emotional expressions persistent, quantifiable, and publicly observable. As Bucher (2018) asserts, reactions such as “likes,” comments, and algorithmic responses function as embodied signals that communicate to adolescents the social value of their emotional expressions. These signals contribute to the shaping of their self-perception and relational expectations. Through repeated interaction with these feedback mechanisms, adolescents develop the ability to regulate their emotional behavior, adapting it to the affective norms inherent in digital platforms. Emotional embodiment emerges as a process negotiated between bodily sensations, technological mediation, and social recognition, in which the body becomes a relational node distributed between subjective experience and digital infrastructure (Dolezal, 2020). Concurrently, digital embodiment provides adolescents with environments conducive to emotional experimentation and identity exploration. The utilization of avatars and mediated representations facilitates the articulation of emotions that are frequently repressed or stigmatized in offline environments. This phenomenon engenders opportunities for individuals to delve into themes such as vulnerability, belonging, and alternative self-narratives (Turkle, 2011; Taylor, 2018). This dimension underscores the ambivalence of digital emotional embodiment, situated between emancipatory potential and the risk of affective normalization. Consequently, it becomes imperative to adopt a critical approach that can effectively grasp the socio-technical tensions inherent in the adolescent emotional experience. Research conducted on virtual environments suggests that digital embodiment can enhance emotional engagement and influence self-perception, thereby highlighting the transformative potential of technology-mediated emotional experience (Gall et al., 2021; Zimmermann et al., 2023). For adolescents, such environments can function as laboratories for emotional development, where affective styles and identities are experimented with and negotiated. However, the potential of digital emotional embodiment is inseparable from its limitations. The use of standardized symbolic repertoires, such as emojis or predefined reaction options, can reduce the richness and nuance of emotional expression or even diminish certain emotions in the eyes of others. Additionally, the systematic arrangement of visibility and feedback has the potential to promote certain emotional tendencies, thereby contributing to the acceptance of particular affective patterns. This dynamic has the potential to influence adolescents’ emotional self-understanding over time. In doing so, it reinforces expectations regarding which emotions are considered acceptable, valuable, or worthy of attention. Digital emotional embodiment prompts inquiries into the authenticity and regulation of emotions. Notable cases include Haboo and Second Life (now conceptually outdated) or Decentraland (a virtual world based on cryptocurrencies). In the context of emotional experiences mediated by digital interfaces and evaluated through engagement metrics, the boundary between spontaneous emotional experience and performative expression is gradually blurring. Adolescents may experience an ongoing tension between expressing emotions as they are subjectively experienced and adapting emotional expressions to anticipated social or algorithmic responses, such as likes, comments, or visibility signals (Papacharissi, 2015; Bucher, 2018). This tension is not exclusively a matter of individual psychology, but rather, it more broadly reflects the socio-technical conditions that structure the development of emotional life in digital environments. From a behavioral science perspective, these dynamics suggest that emotional regulation in adolescence cannot be fully understood without considering the embodied, relational, and technologically mediated nature of digital interaction (Gross, 2015; Kappas, 2011). Emotional regulation is no longer considered exclusively as an intrapsychic process of self-control or cognitive restructuring. Instead, it is increasingly understood as the ability to navigate technological environments that actively respond to emotional states and help shape their expression and intensity. During this process, adolescents not only internalize interpersonal norms and social expectations, but also assimilate the affective logics embedded in digital platforms and artificial intelligence-based systems. These systems include the valorization of emotional intensity, visibility, and immediate reactivity (Beer, 2017; van Dijck et al., 2018). Repeated interaction with systems that reward specific emotional styles contributes to the formation of affective repertoires that are progressively aligned with technological affordances. These repertoires influence the way emotions are recognized, regulated, and communicated. From this perspective, digital emotional embodiment emerges as a crucial component of contemporary adolescence, as it allows us to grasp the dynamic intersection between body, technology, and social interaction. Emotional experience cannot be traced back to a purely bodily dimension or an exclusively symbolic dimension. Rather, it is configured as a process situated in hybrid environments, in which bodily sensations, technological mediation, and social recognition are mutually co-constructed (Dolezal, 2020; Hansen, 2006). This perspective underscores the necessity to transcend conventional dichotomies between online and offline emotional life, acknowledging that adolescents’ emotional development unfolds within integrated ecosystems, wherein digital and physical experiences are profoundly intertwined. This comprehensive process of emotional datafication is not confined to digital communication platforms; it also encompasses the statistical analysis of physiological signals. In this sense, the datafication of physiological signals illustrates how embodied affective states can be rendered legible to digital systems, a process that is increasingly relevant to adolescents’ emotional experience in AI-mediated environments. In the domain of behavioral health research, for instance, the use of physiological indicators such as heart rate variability is increasingly combined with statistical and computational methods to identify behavioral and affective patterns. The application of statistical feature extraction techniques to heart rate signals demonstrates how bodily processes can be translated into quantifiable indicators of emotional and behavioral states (Zhao et al., 2025). These approaches exemplify the broader logic of datafication through which embodied signals become measurable and operational within data-driven environments. These datasets can then be subjected to analysis, facilitating the inference of behavioral and affective dynamics. These approaches exemplify the broader logic of datafication, a term coined by the philosopher Michel Foucault, through which embodied signals become quantifiable indicators within contemporary data-driven research environments. A comprehensive understanding of emotional regulation in adolescence necessitates an approach that considers the behavioral, bodily, and socio-technical dimensions of emotional experience. This multifaceted approach enables a more nuanced understanding of the processes of adaptation, agency, and vulnerability that characterize emotional growth in the digital context.

## 6. The Transformation of Empathy in AI-Mediated Environments

As emphasized by Auriemma (2023b), empathy, defined as the ability to recognize, understand, and respond to the emotional states of others, plays a pivotal role in the emotional and social development of adolescents. In the domain of behavioral sciences, empathy is frequently conceptualized as a cognitive and affective competence, which can be measured at the individual level. This concept is associated with prosocial behavior and emotional intelligence. Despite the fact that this perspective has yielded pertinent empirical insights, it appears to conceptualize empathy as a relatively stable personal attribute. This approach does not sufficiently consider the social and technological conditions under which empathic processes emerge and evolve. From a sociological perspective, empathy is not merely an individual predisposition; rather, it is a relational and situational process shaped by interactional contexts, symbolic signals, and cultural norms. In face-to-face interactions, the basis of empathic understanding is embodied co-presence, which involves gestures, facial expressions, tone of voice, and bodily attunement (Decety & Jackson, 2004). Consequently, empathic processes are increasingly mediated by technological infrastructures that reorganize the way emotions are perceived and interpreted (Collins, 2004). In AI-mediated environments, this transformation is particularly salient. Indeed, artificial intelligence systems play an active role in shaping empathic encounters by filtering, prioritizing, and framing emotional content, as demonstrated by dating applications. Recommendation algorithms influence the emotional narratives to which adolescents are exposed, while emotionally responsive technologies, such as chatbots or adaptive interfaces, simulate empathetic responses through predefined affective models. A prime example of this phenomenon is ChatGPT, which has been shown to produce emotionally positive responses with high consistency. Such systems not only facilitate empathic interaction but also contribute to the redefined conception of empathic understanding by translating emotional signals into data and predictive models (Scribano & Mairano, 2021). This phenomenon can be conceptualized as the virtualization and datafication of empathy, signifying an escalating reliance on algorithmic mediation for empathetic engagement, superseding the traditional mechanisms of direct bodily resonance. To elaborate further, it can be posited that we are witnessing a decline in empathy, wherein digitally mediated communication leads to a reduction in the depth of interpersonal emotional engagement. As an alternative hypothesis, we propose the substitution model observed in the field of artificial intelligence (AI). In this model, algorithmic systems endeavor to simulate or replicate empathetic responses through the computational analysis of emotional signals. In other contexts, a perspective that emphasizes integration can be observed, highlighting how human empathic processes are increasingly intertwined with technological mediation in online interactions—for example, in fitness apps. In this article, a fourth perspective is adopted, whereby these developments are conceptualized as a process of transformation rather than as mere erosion or replacement. Digital infrastructures and AI systems do not eliminate empathy; rather, they contribute to the transformation of the manner in which empathic signals are expressed, interpreted, and disseminated in socio-technical environments. In this sense, empathy becomes partially “datafied,” as emotional expressions are translated into digital traces, metrics, and algorithmically processed signals, while remaining rooted in embodied and socially situated emotional experiences. Adolescents learn to recognize emotions through platform-specific signals and metrics, such as engagement levels or number of reactions. These serve as indicators of emotional relevance, becoming a sort of television “share” for adolescents’ experiences. It has been demonstrated that, over time, these signals have the capacity to influence empathic attention, thereby directing individuals toward emotionally intense content that reflects the platform’s logic of visibility and engagement (Turkle, 2017). Concurrently, the advent of artificial intelligence has given rise to novel forms of emotional connection, thereby enabling adolescents to engage in empathic interaction with distant individuals, fictional narratives, or communities that might otherwise be inaccessible in non-digital settings. For specific populations, particularly those living in conditions of social marginalization, such places can function as spaces for emotional identification and a sense of belonging. However, it is imperative to acknowledge that these prospects coexist with the risks associated with emotional simplification and standardization. When empathetic responses are mediated by predefined emotional categories or algorithmic predictions, the complexity and ambiguity of the emotional experience can be reduced. The evolution of empathy within artificial intelligence-mediated environments exerts a significant influence on emotional regulation. Adolescents who interact with systems that respond to emotional signals may develop a reliance on external feedback to guide their emotional understanding and response. Such dependence has the potential to facilitate emotional learning in specific contexts; however, it can also contribute to the development of forms of emotional dependence, particularly when emotionally responsive technologies are perceived as reliable or non-judgmental interlocutors. In this regard, it is noteworthy that recent surveys reported in the media and in public debates suggest that a significant percentage of teenagers report having emotionally meaningful interactions with conversational AI systems. For instance, a frequently cited statistic asserts that approximately 26% of adolescents worldwide regard ChatGPT as their “most trusted companion.” The research was conducted by CSA Research on behalf of Save the Children for Italy in 2025 and by Common Sense Media for the United States. The results of the research were published in a report titled “Talk, Trust, and Trade-Offs: How and Why Teens Use AI Companions” (Common Sense Media, 2025). However, it is imperative to exercise caution when interpreting these figures. The veracity of survey-based claims is often contingent upon the efficacy of sampling strategies, the manner in which survey questions are worded, and the interpretive ambiguity of relational expressions such as “best friend.” These expressions may elicit playful, ironic, or metaphorical responses rather than stable emotional bonds. From a sociological perspective, these statistics should therefore be understood not so much as precise measurements of emotional relationships with AI, but rather as indicators of broader cultural narratives surrounding young people’s engagement with digital technologies. Instead of accepting these figures at face value, this study employs them as a point of departure to reflect on how emotionally meaningful interactions with digital systems are increasingly framed and discussed in contemporary public discourse. The research findings indicate that a considerable proportion of adolescents utilize ChatGPT and other AI chatbots as companions or confidants. In Italy, approximately 41.8% of adolescents, or four in ten, utilize artificial intelligence for emotional comfort, relationship advice, or support in moments of loneliness. It is noteworthy that a significant proportion of adolescents in the United States have adopted AI-powered chatbots for companionship, friendship, or guidance. Moreover, regular utilization of these technologies is observed, with more than half of the users reporting a frequency of at least two times per month. A survey reveals that 26% of Generation Z consider AI to be a “friend,” while 16% use it specifically as a “psychologist.” Research conducted by Save the Children in 2025 found that approximately 20% of adolescents prefer communicating with a chatbot rather than a human (Save the Children, 2025). In such circumstances, it is not surprising that empathy is partially outsourced to technological systems, which raises questions about emotional autonomy and self-regulation. It is imperative to emphasize that these dynamics should not be interpreted as a rudimentary decline or deterioration of empathy. Rather, they should be understood as a transformation of relational and interaction processes. Indeed, evidence suggests that socio-technical conditions can lead to a reconfiguration of empathic processes (Turkle, 2011). Empathy in AI-mediated environments, therefore, functions at the intersection of embodied experience, symbolic mediation, and algorithmic governance. Adolescents interact with these complex forms of mediation as an integral part of their emotional development, integrating digital signals into their empathetic repertoire. From a behavioral science perspective, this suggests a paradigm shift from a conception of empathy as an individual capacity to a more complex view that interprets it as a distributed process emerging from interactions between individuals and technological systems. This transition is of particular pertinence in the context of adolescent development, a period marked by the maturation of empathic capacities that are susceptible to environmental influences. Acknowledging the socio-technical formation of empathy enables a more nuanced evaluation of the benefits and vulnerabilities associated with AI-mediated emotional environments.

## 7. Opportunities and Risks of the Digitization of Emotions

Emotional embodiment processes are intricate and inherently ambiguous, with their particulars undergoing transformation within digital contexts. These processes offer novel opportunities for emotional development, affective expression, and social connection. However, they concomitantly introduce risks that can affect emotional regulation, psychological well-being, and forms of vulnerability (Steinberg, 2014). Comprehension of this ambivalence necessitates the supersession of deterministic narratives that portray digital technologies as either inherently beneficial or deleterious. It also necessitates the supersession of other narratives belonging to biological determinism, which regard emotions as innate virtues. Adopting a socio-technical perspective that considers the material, symbolic, and organizational conditions underpinning emotional experiences can facilitate the emergence of novel elements and knowledge processes pertinent to the digitization of lifelong learning (Orlikowski, 2007; Couldry & Hepp, 2017). From an opportunity perspective, digital environments have the potential to expand the emotional repertoire of adolescents by providing spaces for expression, experimentation, and recognition. Online platforms, in fact, offer the possibility of expressing emotions that might be complex to express in face-to-face interpersonal contexts, through alternative modes of communication based on images, symbols, texts, and visual narratives (Papacharissi, 2015). For individuals who encounter forms of social marginalization, these environments can function as spaces for emotional inclusion, fostering connections based on shared experiences and emotional affinities rather than physical proximity or social status (Boyd, 2014; Baym, 2015). From a behavioral science perspective, these dynamics can promote emotional learning by facilitating processes of reflection, perspective-taking, and social engagement. Digital technologies have the potential to function as resources for emotional regulation and well-being, particularly through emotionally responsive systems, such as AI-based support tools or adaptive educational platforms (De Jaegher et al., 2010). These systems have the capacity to provide feedback and guidance, which can be instrumental in the development of emotional awareness and coping strategies, particularly within educational or preventive contexts (Calvo & Peters, 2014). In circumstances characterized by limited access to conventional support services, such technologies have the potential to promote the acquisition of emotional skills and resilience, provided that technological mediation is critically designed and appropriately contextualized. Concurrently, the risks associated with the digitalization of emotions are considerable and distributed unequally. Consequently, there is a pressing need for digital literacy, a concept that has been repeatedly emphasized yet remains largely unimplemented. A significant critical concern pertains to the standardization of emotional expression, which is driven by the affordances of social media platforms and the underlying algorithmic logics that prioritize certain affective styles over others (Gillespie, 2018). When emotional visibility and recognition are closely linked to engagement metrics, adolescents may adapt their affective expressions to align with platform norms, progressively narrowing the range of socially legitimate emotions. This dynamic has the potential to influence emotional self-understanding in the long term, thereby hindering the recognition and articulation of complex or ambivalent emotional states. A notable additional risk is emotional dependence and emotional dysregulation. The continuous interaction with emotionally reactive technologies has been demonstrated to enhance dependence on external feedback for the validation and regulation of emotions. A 2020 behavioral study by Odgers and Jensen revealed a correlation between these dynamics and increased anxiety, stress, and reduced emotional autonomy. This finding underscores the need to analyze how socio-technical environments structure affective regulation processes (Odgers & Jensen, 2020). The advent of digital technologies has given rise to concerns regarding emotional exposure and the phenomenon of overstimulation. Algorithmic systems have a tendency to prioritize emotionally salient content, thereby amplifying affective intensity and reducing opportunities for emotional recovery (Citton, 2017). Prolonged exposure to emotionally stimulating content has been demonstrated to have a significant impact on the emotional reactivity of adolescents. This exposure has been shown to contribute to an increase in emotional reactivity and difficulties in managing affective responses. From a sociological perspective, such outcomes should not be interpreted as expressions of individual fragility. Rather, they should be understood as structural effects of digital environments designed to maximize engagement and attention (Citton, 2017). It is imperative to underscore the manner in which the potential risks associated with the digitization of emotions are intricately intertwined with broader social inequalities. Access to digital resources, levels of digital and emotional literacy, and socio-economic conditions exert a significant influence on how adolescents experience and interpret digital emotional environments (Livingstone & Helsper, 2007). In the absence of adequate educational and institutional support, the most vulnerable populations may be disproportionately affected by the effects of emotional datafication and algorithmic governance. The aforementioned dynamics underscore the necessity for integrated responses at the educational and political levels. In the scientific realm, it is imperative to transcend the confines of developing individual competencies. Instead, there is a necessity to contemplate the environmental factors that influence emotional experiences (Livingstone & Helsper, 2007). Educational strategies aimed at fostering emotional competence should be complemented by the incorporation of digital emotional literacy practices. These practices facilitate in adolescents the development of critical understanding regarding the manner in which emotions are mediated, quantified, and oriented by digital systems and artificial intelligence (Gillespie, 2018). At the policy level, the digitization of emotions necessitates the implementation of governance measures informed by the principles of ethical design. This governance must encompass transparency in the processes of algorithmic decision-making and the protection of adolescents’ emotional data (Floridi et al., 2018). Policies that acknowledge emotions as socially and technologically integrated processes can facilitate the creation of digital environments that promote emotional well-being rather than exploit affective vulnerability. To conclude, the opportunities and risks of the digitalization of emotions are inextricably linked. While such processes can promote emotional development and inclusion, they can also reinforce emotional standardization, dependence, and vulnerability. As Nicolescu (2002) noted, confronting these challenges necessitates a transdisciplinary approach that can effectively integrate behavioral perspectives, sociological analysis, and political orientations. This approach is crucial for overcoming the disciplinary fragmentation that persists in much of the research on this subject.

## 8. Discussion and Conclusions

The bibliometric results obtained from this study corroborate the theoretical considerations that were developed in this article. The prevailing orientation of research on clusters towards psychology, propelled by technological advancement, coupled with the relative marginality of sociological and embodiment-centered perspectives, mirrors a persistent conceptual fragmentation in the study of digital emotions. The fragmentation of emotions is a phenomenon that helps explain why such emotions are often analyzed as individual psychological variables or as computable signals, with limited attention to their embodied and socio-technical constitution. From this standpoint, bibliometric analysis is not presented as an independent empirical contribution, but rather as a contextual and reflective instrument that substantiates the necessity for an integrative theoretical framework, such as that of digital emotional embodiment proposed in this study. The theoretical contribution of the article lies precisely in this reconceptualization. The framework of digital emotional embodiment functions as an analytical instrument capable of integrating behavioral sciences, the sociology of emotions, and digital media studies, thereby transcending methodologies that interpret emotions as predominantly individual outcomes of technological utilization. Conversely, emotional development, particularly during adolescence, is conceptualized in this framework as a process structured by the environment and mediated socio-technically. In this perspective, fundamental concepts such as emotional regulation and empathy are reinterpreted, emphasizing their influence not only by individual competencies but also by the architectures of digital platforms, algorithmic feedback mechanisms, and data-based norms that regulate visibility and emotional validation. This theoretical shift entails a change in analytical focus, moving from the effects of digital technologies on emotions to the socio-technical conditions within which emotional dynamics are constituted and stabilized in contemporary digital societies. The present article endeavors to furnish an explanation of the processes of digitization of emotions in adolescence, with particular consideration for the phenomena of emotional embodiment and empathy in environments mediated by artificial intelligence. It is imperative to acknowledge that the study’s objective does not entail the empirical measurement of adolescents’ emotional experiences or the evaluation of specific digital platform effects. Instead, the primary contribution of this study is the development of a theoretical framework that serves as a guide and source of information for future empirical research. From this standpoint, bibliometric analysis assumes an exploratory and contextual function. The objective of this analysis is to map the structure of the extant literature and to identify recurring themes, dominant orientations, and areas of theoretical marginality. This analysis underscores the conceptual lacunae inherent in the study of digital emotions, particularly the inadequate incorporation of sociological and bodily dimensions within a field predominantly dominated by psychological and computational approaches. Bibliometrics provides no autonomous empirical contribution; rather, it serves to reinforce the theoretical underpinnings of the work in question. This underscores the necessity for future qualitative, quantitative, or mixed studies that are capable of empirically investigating the socio-technical processes that structure adolescent emotional experience in digital environments. The present analysis conceptualizes emotions as socially rooted, embodied, and technologically mediated processes. It draws on the author’s categorical work from 2023, with the aim of filling a significant gap within the behavioral sciences. Emotional phenomena are often examined at the individual level or reinterpreted in computational terms, with limited consideration of the socio-technical conditions that shape emotional experience (Auriemma, 2023b; Lupton, 2018a; Rose, 2019). A seminal insight of this study was the reinterpretation of the digitization of emotions as a structural condition of contemporary adolescence, challenging the prevailing interpretation of emotions as a peripheral or contingent phenomenon. In the context of digital environments, emotions are not merely expressed or communicated through technology; rather, they are actively manipulated by platform logic, algorithmic mediation, and emotionally reactive systems (Gillespie, 2018; Zuboff, 2019). This perspective has the potential to broaden the scope of existing behavioral research by demonstrating how emotional regulation, empathic engagement, and psychological well-being are increasingly influenced by environmental factors embedded in digital infrastructures. The concept of digital emotional embodiment provides a novel analytical framework for understanding adolescents’ emotional experiences in mediated contexts. The analysis demonstrates a substantial correlation between bodily experience and technological mediation, thereby challenging the prevailing dichotomies between online and offline emotions that are entrenched in a significant portion of the scientific literature (Damasio, 1999; Pink, 2011). Emotional embodiment in digital environments is a multifaceted phenomenon that can be conceptualized as a hybrid process, wherein bodily sensations, symbolic representations, and algorithmic feedback are intricately intertwined. From the perspective of behavioral sciences, this implies the need to consider embodiment not only as a biological or sensorimotor process but also as a socially and technologically situated phenomenon (Gallagher, 2017). In a similar vein, the examination of empathy within artificial intelligence-mediated environments contributes to contemporary discourses by transcending the limitations of conventional, trait-based, or exclusively cognitive conceptualizations. In this context, empathy is reconceptualized as a distributed and mediated process, shaped by socio-technical systems that organize emotional visibility, attention, and modes of interaction (De Jaegher et al., 2010; Baym, 2015). While AI-driven environments have the potential to foster novel forms of emotional connection and perspective-taking, they concomitantly introduce risks related to emotional standardization, the datafication of affective experience, and dependence on algorithmic feedback (Illouz, 1997; Zuboff, 2019). Awareness of these dynamics facilitates a more nuanced understanding of empathic development in adolescence, capable of considering both enabling factors and structural constraints. A discussion of the opportunities and risks associated with the digitalization of emotions reveals the inherent ambivalence of this process. Digital environments have the potential to facilitate emotional expressiveness, identity experimentation, and social inclusion, particularly among adolescents who encounter challenges in offline relational contexts (Boyd, 2014). Conversely, the standardization of emotional evaluation, predicated on engagement metrics and continuous emotional exposure, has the potential to compromise emotional autonomy and psychological well-being. It is imperative to underscore that these risks cannot be ascribed purely to individual vulnerabilities. Rather, they must be construed as consequences of socio-technical configurations devised to optimize visibility, attention, and engagement (Couldry & Mejias, 2019). From an applied perspective, the results of the present study have important implications for education, social intervention, and public policy. In the scientific community, particularly within the domain of behavioral sciences, there is an escalating cognizance of the significance of environmental factors in influencing emotional behavior. The theoretical framework delineated herein aims to consolidate this transition by underscoring the necessity to integrate digital emotional literacy as a fundamental component of adolescent education. This literacy is not confined to individual emotional awareness but encompasses a critical understanding of the role of digital platforms and artificial intelligence systems in mediating, orienting, and evaluating emotional experience (Livingstone et al., 2017). Educational interventions that incorporate this perspective have proven to be more effective in promoting emotional regulation, resilience, and critical agency in digital environments. At the policy level, the digitization of emotions necessitates the implementation of emotionally informed approaches to the governance of AI (artificial intelligence) and digital platforms. To ensure the emotional well-being of adolescents, it is imperative to extend the scope of concern beyond the realms of personal data protection and content moderation, encompassing the affective architectures of digital technologies (Yeung, 2018). Policies oriented toward algorithmic transparency, ethical design, and the accountability of emotionally responsive technologies have the potential to contribute to the construction of digital environments that support healthy emotional development rather than exploit affective vulnerability. Notwithstanding the theoretical contributions proffered, this analysis is encumbered by inherent limitations. As a conceptual study, it does not provide direct empirical evidence on adolescents’ experiences in relation to digital emotional embodiment or AI-mediated empathy. It is imperative that future research address these processes through an empirical approach, combining behavioral measures with qualitative and sociological methodologies. Longitudinal studies have been identified as a particularly promising method for understanding the influence of digitized emotional environments on emotional development over time. In conclusion, the article posits that addressing adolescent emotional development in the digital age necessitates a shift from models that are exclusively focused on the individual. Instead, there is a need to adopt theoretical frameworks that recognize emotions as embodied, relational, and socio-technically mediated processes. The integration of perspectives from the sociology of emotions, embodiment theory, and behavioral sciences allows for the development of a comprehensive approach to understanding the digitization of emotions in adolescence. This approach should be regarded as a contribution to the theoretical debate, while also providing a solid foundation for educational and policy strategies aimed at promoting emotional well-being in societies that are increasingly digitized and driven by artificial intelligence. The concept of digital emotional embodiment offers a transferable analytical lens for examining emotional development in different AI-mediated contexts. This concept provides a basis for future empirical research and emotionally informed educational and policy interventions.

## Data Availability

All analyses and results were carried out using Bibliometrix 5.0 software and are therefore stored in my private archive. If necessary and requested, I can provide the data contained in the article.
